# *De novo* construction of an expanded transcriptome assembly for the western tarnished plant bug, *Lygus hesperus*

**DOI:** 10.1186/s13742-016-0109-6

**Published:** 2016-01-28

**Authors:** Erica E. Tassone, Scott M. Geib, Brian Hall, Jeffrey A. Fabrick, Colin S. Brent, J. Joe Hull

**Affiliations:** Plant Physiology and Genetics Research Unit, US Arid Land Agricultural Research Center, USDA Agricultural Research Services, Maricopa, AZ USA; Tropical Crop and Commodity Protection Research Unit, Daniel K Inouye Pacific Basin Agricultural Research Center, USDA Agricultural Research Services, Hilo, HI USA; Department of Plant and Environmental Protection Sciences, University of Hawaii Manoa, Honolulu, HI USA; Pest Management and Biocontrol Research Unit, US Arid Land Agricultural Research Center, USDA Agricultural Research Services, Maricopa, AZ USA

**Keywords:** Transcriptome, *Lygus hesperus*, Plant bug, Miridae, RNA-Seq, Trinity

## Abstract

**Background:**

The plant bug *Lygus hesperus* Knight is a polyphagous pest of many economically important crops. Despite its pest status, little is known about the molecular mechanisms responsible for much of the biology of this species. Earlier *Lygus* transcriptome assemblies were limited by low read depth, or because they focused on specific conditions. To generate a more comprehensive transcriptome, we supplemented previous datasets with new reads corresponding to specific tissues (heads, antennae, and male reproductive tissues). This transcriptome augments current *Lygus* molecular resources and provides the foundational knowledge critical for future comparative studies.

**Findings:**

An expanded, Trinity-based *de novo* transcriptome assembly for *L. hesperus* was generated using previously published whole body Illumina data, supplemented with 293 million bp of new raw sequencing data corresponding to five tissue-specific cDNA libraries and 11 Illumina sequencing runs. The updated transcriptome consists of 22,022 transcripts (average length of 2075 nt), 62 % of which contain complete open reading frames. Significant coverage of the BUSCO (benchmarking universal single-copy orthologs) dataset and robust metrics indicate that the transcriptome is a quality assembly with a high degree of completeness. Initial assessment of the new assembly’s utility revealed that the length and abundance of transcripts predicted to regulate insect physiology and chemosensation have improved, compared with previous *L. hesperus* assemblies.

**Conclusions:**

This transcriptome represents a significant expansion of *Lygus* transcriptome data, and improves foundational knowledge about the molecular mechanisms underlying *L. hesperus* biology. The dataset is publically available in NCBI and GigaDB as a resource for researchers.

## Data description

### Background

The western tarnished plant bug *Lygus hesperus* Knight is a polyphagous pest with an extensive host plant range including many economically important food, fiber, and seed crops [1]. While control measures have traditionally relied on broad-spectrum insecticides, negative ecological ramifications and evolving insecticide resistance have reduced the continued viability of this approach. As a consequence, there is growing interest in biorational-based strategies; however, the development of such approaches requires a comprehensive understanding of a species’ underlying biology. Towards this end, we previously reported on the sequencing and assembly of two *L. hesperus* transcriptomes: a general Roche 454-based assembly [2], and a second Illumina-based assembly incorporating sequence information from adults under thermal stress [3]. Those databases were developed using sequence data derived from whole bodies. Although this approach yields substantial data, whole body analysis tends to mask underrepresented genes that are expressed primarily in specific tissues or under specific conditions. To generate a more comprehensive transcriptome, here we supplement our previous thermal dataset with reads from specific tissues: heads, antennae, and male reproductive tissues. Incorporation of these new datasets expands the current *L. hesperus* database, provides greater depth of coverage, and enables new research for the better understanding of *Lygus* biology.

### Samples

All samples and tissues were derived from an *L. hesperus* laboratory colony maintained at the United States Department of Agriculture-Agricultural Research Service (USDA-ARS) Arid Land Agricultural Research Center (ALARC) in Maricopa, Arizona, USA. The colony was reared at 27–29 °C under 20 % humidity with an L14:D10 photoperiod, and fed an artificial diet [4]. Nymphs and adults used for RNA preparation were from eggs deposited in agar oviposition packets and maintained as described previously [5]. Our initial Illumina-based transcriptome [3] was generated using 10-day old adults exposed for 4 h to one of three temperatures (4 °C, 25 °C, or 39 °C). To provide deeper coverage of transcripts encoding proteins functioning in olfaction, central nervous system-mediated behaviors, and male reproduction, sex-specific antennae, heads, and male accessory glands were dissected and stored at −20 °C in RNALater (Ambion/Life Technologies, Carlsbad, CA). The antennae samples represent ~500 unmated 7–9-day old adult males, and ~600 unmated 7–9-day old adult females. Heads (8–12 per stage/age per replicate) without antennae were collected across three biological replicates from 3rd instar nymphs, 4th instar nymphs, late 5th instar nymphs, and unmated adults of both genders at 1, 3, 7, 10, and 15 days post-eclosion. Accessory glands (30 per replicate) were dissected in phosphate-buffered saline from 7 to 8-day-old adult males 24 h post-mating and from similarly aged unmated cohorts. Total RNA extraction and library generation (TruSeq RNA Sample Preparation Kit v2; Illumina Inc., San Diego, USA) were performed as described previously [3] at the University of Arizona Genomics Center. All samples were sequenced using an Illumina HiSeq2000 or HiSeq2500 in Rapid Run mode (paired-end 100-bp reads).

### Data filtering

Approximately 438 million reads were obtained, resulting in over 257 GB of 2 x 100 bp paired-end data. Raw read quality was assessed and filtered with a custom pipeline using FastQC (V 0.10.1) and Trimmomatic (V 0.32), using the parameters ILLUMINACLIP:TruSeq3-PE.fa:2:30:10 LEADING:10 TRAILING:20 SLIDINGWINDOW:4:25 MINLEN:36 to remove adapter sequences and filter by quality score. Short read archive (SRA) accessions for all data are found in Table 1.

**Table 1:** Accession numbers for *L. hesperus* sequence reads and assembled transcripts

Sample	Short Read Archive	BioSample	BioProject
10-day-old adults^a^			
4 °C	SRX483635, SRX483674, SRX483877	SAMN02679940-42	PRJNA238835
25 °C	SRX483950, SRX484037, SRX484042	SAMN02679943-45	"
39 °C	SRX484076, SRX484077, SRX484079	SAMN02679946-48	"
Antennae			
Male	SRX317887, SRX317888	SAMN02222162-63	PRJNA210219
Female	SRX317885, SRX317886	SAMN02222160-61	"
Accessory Gland			
Mated	SRX318362, SRX318363	SAMN02222164-65	PRJNA210220
Unmated	SRX318364, SRX318365	SAMN02222166-67	"
Head	SRX1072689, SRX1155625, SRX1155629	SAMN03792993-95	PRJNA284294

^a^Data from Hull et al. 2014 [3]

### Transcriptome assembly

Data used for assembly corresponded to the ~145 million bp of sequence reads generated previously [3], and 293 million bp of new data from 11 Illumina runs covering five tissue-specific libraries. Prior to assembly, the four datasets (thermal-based, head, antennae, and accessory gland) were concatenated, and read abundance was normalized to 50X coverage using the *in silico* normalization tool in Trinity to improve assembly time and minimize memory requirements. Filtering and normalization reduced the dataset to 15 Gb, comprising approximately 32 million normalized read pairs, which were then assembled using default parameters in Trinity (r2014_07-17). Transcript expression levels were estimated with RSEM [6] and open reading frames (ORFs) were predicted using Transdecoder [7]. Hmmer3 was used to identify additional ORFs matching Pfam-A domains. Following transcriptome assembly, reads were filtered, sorted, and prepared for NCBI transcriptome shotgun assembly (TSA) submission as previously described [8].

### Annotation

Functional annotation was performed at the peptide level using a custom pipeline [8] that defines protein products and assigns transcript names. Predicted proteins/peptides were analyzed using InterProScan5, which searched all available databases including Gene Ontology (GO) [9]. BLASTp analysis of the resulting proteins was performed with the UniProt Swiss Prot database (downloaded 11 February 2015). Annie [10], a program that cross-references SwissProt BLAST and InterProScan5 results to extract qualified gene names and products, was used to generate the transcript annotation file. The resulting .gff3 and .tbl files were further annotated with functional descriptors in Transvestigator [8].

### Quality, completeness and depth of the comprehensive *L. hesperus* transcriptome

To assess the relative quality and completeness of our assembly, we compared core statistics for published *Lygus* transcriptomes [2, 3, 11] with those of the *L. hesperus* transcriptome described in this study (Table 2). The total number of sequence reads used in the current assembly represent 1660 and 300-fold increases over those used in the *L. lineolaris* transcriptome [11] and the initial Roche 454-based *L. hesperus* transcriptome [2] respectively. The expansion of read inputs resulted in average transcript lengths increasing from 725 to 2075 bp, and a larger percentage of transcripts with BLAST hits and assigned GO terms. Compared with the previously published Illumina transcriptome, inclusion of nearly three times the number of reads had little effect on average transcript length, and only marginally increased the N50 for the longest transcript per unigene (Table 2). However, low abundance isoforms were specifically removed during data normalization in the expanded assembly, a process that was modified from that used in the construction of the previous Illumina assembly. Consequently, while the expanded assembly represents less overall “gene space” than the previous assembly, it likely provides a more accurate reflection of the transcript landscape. More importantly, the expanded dataset increases overall coverage of transcripts critical to tissue-specific functions.

**Table 2:** Transcriptome assembly and annotation statistics compared with previous Lygus transcriptomes

		Transcriptome		
	*L. lineolaris* ^a^	*L. hesperus* (454)^b^	*L. hesperus* (thermal)^c^	*L. hesperus* (current)^d^
Assembly				
Total no. read pairs	262,555	1,429,818	144,898,116	437,850,562
Normalized reads (*in silico* normalization)	-	-	16,191,383	32,342,216
Total no. transcripts	6970	36,131	45,706	22,022
Average transcript length	392 (100–3466)	725 (2–13,480)	2237 (300–23,322)	2073 (297–23,350)
Total assembled bases (all transcripts)	-	32,252,977	102,246,199	45,687,929
Total assembled bases (longest transcript per unigene)	-	28.8 Mb	39.8 Mb	31.6 Mb
N50 (all transcripts)	-	2430	2989	2610
N50 (longest transcript per unigene)	-	1849	2638	2726
%GC	-	0.41	0.44	0.45
Proteins with complete ORF (%)	-	-	-	13,689 (62.1 %)
Annotation				
No. transcripts with a BLAST hit	3126 (44.9 %)	19,393 (54 %)	-	16,942 (76.9 %)
No. transcripts with GO term	2196 (31.5 %)	7898 (21 %)	-	12,114 (54.9 %)
PFAM	-	3705 (22.2 %)	-	14,575 (66.1 %)

Data from: ^a^Magalhaes et al. 2013 [11]; ^b^Hull et al. 2013 [2]; ^c^Hull et al. 2014 [3]

The respective *L. hesperus* assemblies were also evaluated using the BUSCO (benchmarking universal single-copy orthologs) arthropod gene set [12], which uses 2675 near-universal single-copy orthologs to assess the relative completeness of genome and transcriptome assemblies. The percentage of conserved genes identified in the new *L. hesperus* assembly compares favorably with metrics reported for a number of insect transcriptomes and model insect genome assemblies (Table 3). Compared with the previous Illumina assembly, BUSCO genes in the new *L. hesperus* assembly were less fragmented, indicating the presence of more full-length sequences. The relatively high number of duplicates identified in the *L. hesperus* assemblies likely reflect isoforms of single unigenes, rather than true gene duplications.

**Table 3:** BUSCO^a^ analysis of assembly completeness

Species	Complete (%)	Duplicated (%)	Fragment (%)	Missing (%)
*L. hesperus* Transcriptomes				
454-based^b^	56	18	13	29
Illumina-thermal^c^	77	43	11	10
Illumina*-*current	74	33	7.3	17
Select Insect Transcriptomes^d^				
*Nilaparvata lugens* (GI:604923024)	64	-	19	15
*Musca domestica* (GI:510208131)	6.4	-	6.3	87
*Spodoptera exigua* (GI:556694752)	73	-	11	14
*Drosophila serrata* (GI:570485056)	8.5	-	21	70
Select Insect Genomes^d^				
*Pediculus humanus* (PhumU2)	92	3.9	6.1	1.6
*Acyrthosiphon pisum* (GCA_000142985.2)	72	6.1	15	12
*Drosophila melanogaster* (Dmel_r5.55)	98	6.4	0.6	0.3

^a^Simão et al. 2015 [13]

^b^Hull et al. 2013 [2]

^c^Hull et al. 2014 [3]

^d^see Supplementary Data [12] for arthropod BUSCO assessments

**Fig. 1:**
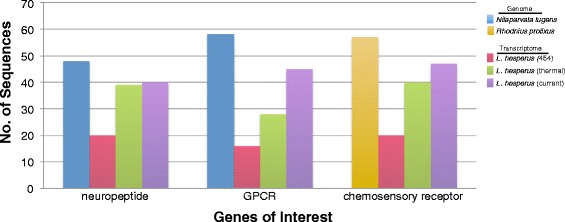
Relative transcript depth of the respective *L. hesperus* transcriptomes. tBLASTx analyses were performed using queries corresponding to genes of interest identified in genome assemblies of *Nilaparvata lugens* or *Rhodnius prolixus*. The *L. hesperus* transcriptomes analyzed include the initial Roche 454-based assembly [2], an Illumina-based thermal assembly [3], and the current assembly. tBLASTx search criteria for the neuropeptide analysis used an e-value of 10^−1^, whereas the G protein-coupled receptor (GPCR) and chemosensory receptor analyses used an e-value of 10^−5^ and transcripts ≥300 nt in length

Next, we used sequences encoding neuropeptides, G protein-coupled receptors, and chemosensory receptors to more fully evaluate the effect of expanding the current assembly with tissue-specific sequencing data. These gene sets mediate much of insect physiology and behavior, and are frequently characterized by spatially restricted expression. The query sequences used in the tBLASTx analyses are from two insect species (*Nilaparvata lugens* and *Rhodnius prolixus*) within the same phylogenetic order (Hemiptera) as *L. hesperus*. The first analysis, which used the 48 neuropeptide sequences reported in *N. lugens* [13] as queries, revealed nearly twice as many homologous sequences in the Illumina-based assemblies as in the initial Roche 454 assembly (Fig. 1). Subsequent searches using *N. lugens* G protein-coupled receptors [13] or *Rhodnius prolixus* chemosensory receptors [14, 15] as queries identified more transcripts ≥300 nt in length in the new, expanded assembly than in the previous transcriptomes. Based on these comparisons, we conclude that the expanded transcriptome represents a marked improvement over the first 454-based assembly, and provides greater coverage of tissue-specific transcripts, such as chemosensory genes and neuropeptide precursors, relative to the previous Illumina assembly. This expanded assembly extends previous work and provides a more comprehensive resource to facilitate the development of new research avenues into the molecular basis of *L. hesperus* biology.

## Availability of supporting data

The filtered and annotated transcriptome was deposited at GenBank as a TSA under the accession GDHC01000000, associated with BioProject PRJNA284294. NCBI accession identifiers for all of the associated SRA, Biosample, and Bioproject data repositories are listed in Table 1. Datasets further supporting the results of this article are available in the *GigaScience* repository, GigaDB [16].

## Abbreviations

ALARC: arid land agricultural research center
BUSCO: bench-marking universal single-copy ortholog
GO: gene ontology
GPCR: G protein-coupled receptor
ORF: open reading frame
SRA: short read archive
TSA: transcriptome shotgun assembly
USDA-ARS: United States Department of Agriculture-Agricultural Research Service
XSEDE: extreme science and engineering discovery environment
